# Bioengineering Methods in MicroRNA-Mediated Direct Reprogramming of Fibroblasts Into Cardiomyocytes

**DOI:** 10.3389/fcvm.2021.750438

**Published:** 2021-10-25

**Authors:** Camilla Paoletti, Valeria Chiono

**Affiliations:** Department of Mechanical and Aerospace Engineering, Politecnico di Torino, Turin, Italy

**Keywords:** myocardial infarction, microRNAs, fibroblasts, cardiomyocytes, cell reprogramming, tissue engineering, nanoparticles

## Abstract

Ischemic heart disease is the major cause of mortality worldwide. Despite the most recent pharmacological progresses, cardiac regeneration is yet not possible, and heart transplantation is the only therapeutic option for end-stage heart failure. Traditional cardiac regenerative medicine approaches, such as cell therapies and tissue engineering, have failed in the obtainment of human functional cardiac tissue, mainly due to unavailability of high quantities of autologous functional cardiomyocytes (CMs), low grafting efficiency, and/or arrhythmic events. Direct reprogramming (DR) of fibroblasts into induced CMs (iCMs) has emerged as a new promising approach for myocardial regeneration by *in situ* transdifferentiation or providing additional CM source for cell therapy. Among available DR methods, non-viral transfection with microRNAs (miRcombo: miR-1, miR-133, miR-208, and miR-499) appears promising for future clinical translation. MiRcombo transfection of fibroblasts could be significantly improved by the development of safe nanocarriers, efficiently delivering their cargo to target cells at the required stoichiometric ratio and overall dose in due times. Newly designed *in vitro* 3D culture microenvironments, providing biomimetic biophysical and biochemical stimuli to miRcombo-transfected cells, significantly increase the yield of fibroblast transdifferentiation into iCMs, enhancing CM gene expression. Epigenetic regulation of gene expression programs, critical to cell lineage commitment, can also be promoted by the administration of specific anti-inflammatory and anti-fibrotic soluble factors, helping in suppressing fibroblast signature. The aim of this mini-review is to introduce the readers to a relatively unknown field of cardiac research integrating bioengineering tools as relevant for the progress of miRNA-mediated cardiac DR.

## Introduction

Ischemic heart disease is a major cause of mortality with more than 23 million cases worldwide (1, 2). During myocardial infarction (MI), billions of cardiomyocytes (CMs) are irreversibly lost and replaced by cardiac fibroblasts (CFs) forming a non-contractile scar tissue, which undergoes continuous remodeling, causing left ventricle dilation and progressive heart failure (3, 4). Given the poor endogenous regenerative potential of the adult heart, recovery of cardiac functionality could be accomplished by the replenishment of lost CMs. However, cell therapies and cardiac tissue engineering strategies have achieved limited success due to poor engrafting, survival, and integration of implanted cells, alone or in combination with biomaterials, into the host tissue and the unmet need for a source of mature and functional CMs (1–4).

Direct reprogramming (DR) of fibroblasts into induced CMs (iCMs) has emerged as a new source for CMs (5–7). Previous literature has reported several different cardiac DR strategies, including the upregulation of cardiac transcription factors (TFs) (8), the administration of complex combinations of small molecules (9), and the modulation of microRNAs (miRNAs) (6). MiRNAs are short non-coding RNAs (of ~21 nucleotides) that regulate gene expression post-transcriptionally (10).

Despite excitement on DR potentialities for cardiac regeneration, the approach is in need of optimization. Main limitations include the low yield of fibroblast DR into iCMs, the wide use of unsafe viral vectors, and the generation of predominantly immature, partially reprogrammed iCMs (11, 12).

**Figure 1:**
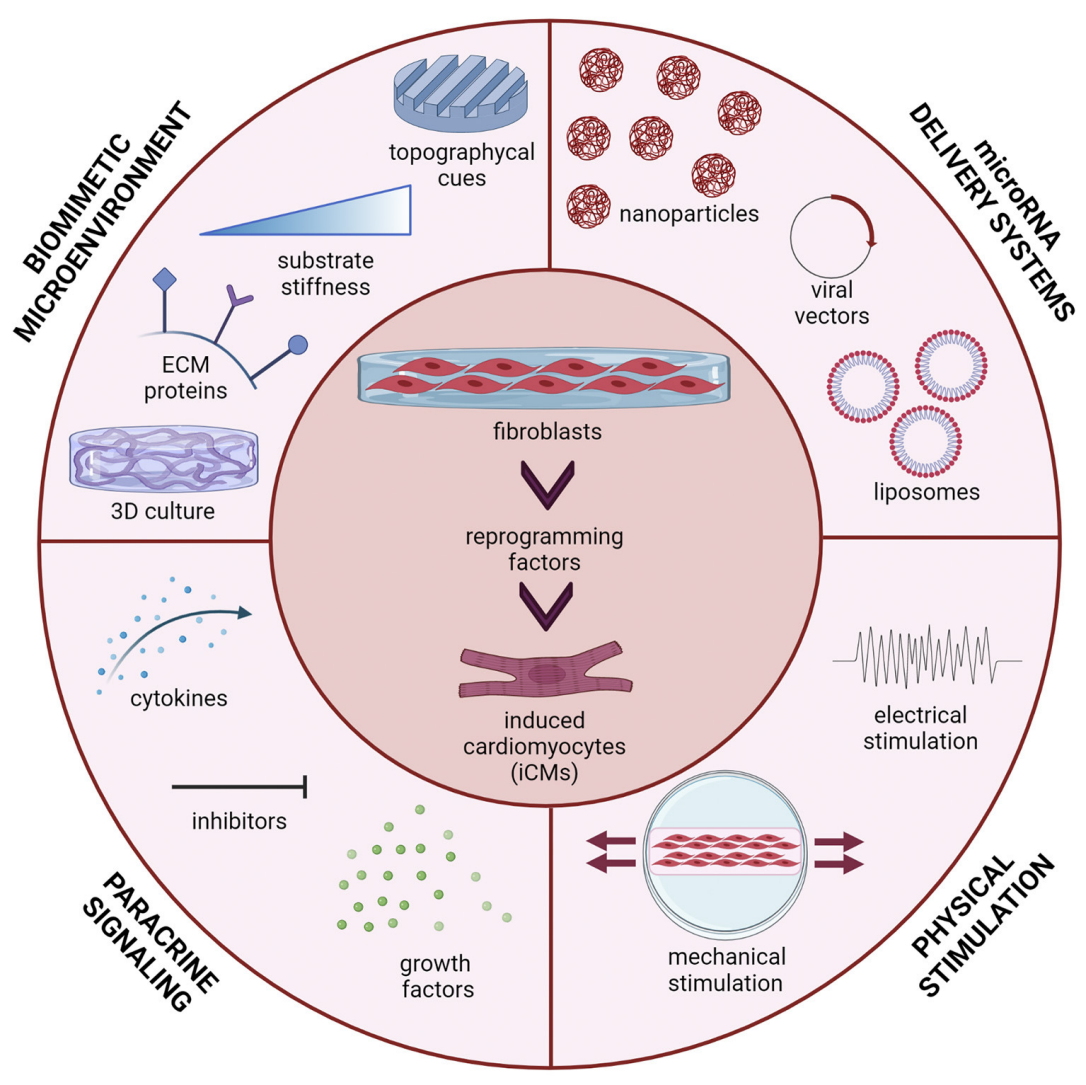
Multiple stimuli can affect direct reprogramming (DR) of fibroblasts into induced cardiomyocytes (iCMs): microRNA delivery strategy (viral vectors, liposomes, polymeric nanoparticles); the microenvironment in which cells are cultured (three-dimensional culture, topographical cues, substrate stiffness and extracellular matrix proteins); paracrine signals (cytokines, inhibitors and growth factors) and physical stimuli (mechanical stretching, electrical stimulation). Figure was created using Biorender.com.

The present mini-review focuses on miRNA-mediated DR of fibroblasts into iCMs as a promising approach for future translation of cardiac DR into clinical settings. Herein, we discuss the key role of bioengineering research in improving cardiac DR efficiency and iCM maturation, through the design of efficient and safe miRNA-releasing nanocarriers and biomimetic *in vitro* culture microenvironments (Figure 1).

## Non-viral MicroRNA Delivery Systems for Direct Reprogramming

### MicroRNA-Mediated Reprogramming of Fibroblasts Into Cardiomyocytes

The use of miRNAs for cardiac DR was first studied in 2012 by Jayawardena et al. (6). An accurate screening of CM-specific miRNAs allowed the selection of six miRNA candidates involved in CM differentiation and development. A minimal combination of four miRNAs called miRcombo (miR-1, miR-133, miR-208, and miR-499) was then identified as able to promote DR of mouse fibroblasts into iCMs (6, 7). The role of such miRNAs in cardiac development was subsequently reported by several studies (13). MiR-1 and miR-133 are co-transcribed in the cardiac and skeletal muscle tissues during embryonic development, and their expression increases until adulthood (13). MiR-1 is involved in regulating CM proliferation and ventricular organization. MiR-133 shares common functions with miR-1. Conversely, miR-208 and miR-499 mostly regulate the expression of α and β isoforms of myosin heavy chain (MHC), which are involved in CM contraction (13). Transient transfection with miRcombo, using a commercial transfection agent (DharmaFECT™), was sufficient to induce *in vitro* DR of mouse neonatal and adult fibroblasts into iCMs (6, 14). Transfected cells expressed CM genes and proteins, showing sarcomeric organization and spontaneous calcium oscillations. Moreover, miRcombo delivery using a lentivirus induced *in vivo* DR of fibroblasts into iCMs (14).

Initially, microRNA delivery and upregulation of cardiac TFs were combined to induce DR of human fibroblasts into iCMs in mixed viral/non-viral approaches (15, 16). A reprogramming cocktail consisting of four TFs (Gata4, Tbx5, Hand2, and Myocardin) and two miRNAs (miR-1 and miR-133) was the most efficient in inducing DR of human foreskin fibroblasts (HFFs), adult human dermal fibroblasts (AHDFs), and adult human CFs (AHCFs) into iCMs (15).

However, in 2020, Paoletti et al. demonstrated that non-viral transient transfection with miRcombo (using DharmaFECT) is enough to trigger the *in vitro* transdifferentiation of AHCFs into iCMs (7). MiRcombo-transfected AHCFs showed enhanced expression of cardiac TFs (Gata4, Mef2c, Tbx5, and Hand2) at 7 days post-transfection, while at 15 days, 11% of cells expressed cardiac troponin T (cTnT), and at 30 days, 38% of cells showed spontaneous calcium oscillations (7).

Recently, miRcombo-mediated cardiac DR efficiency was improved by transfecting mouse fibroblasts with a polycistronic vector, inducing equivalent expression levels of the four microRNAs of miRcombo (17).

Recently, the administration of a polycistronic vector inducing equivalent expression levels of miRcombo was found to improve efficiency of mouse fibroblasts DR into iCMs (17), suggesting the need for delivery vectors, ensuring miRcombo delivery at a stoichiometric ratio.

### Alternative Strategies for MicroRNA Delivery in Direct Reprogramming

In the last years, alternative non-viral strategies to lipid nano-formulations have been proposed for miRNA delivery. Biomaterial-based nanoparticles (NPs) should efficiently encapsulate miRNAs, protecting them from rapid degradation and ensuring their efficient release to target cells, through specific ligands on NP surface for receptor-mediated endocytosis (18). Synthetic polymers [e.g., poly(lactic-*co*-glycolic acid) (PLGA); poly(ethylene glycol) (PEG); and poly(ethylenimine) (PEI)], natural polymers (e.g., chitosan), and inorganic materials (e.g., gold and calcium phosphate) have been investigated as biomaterials for miRNA-loaded nanocarriers (18). Muniyandi et al. have proposed PLGA/PEI NPs encapsulating miR-1 and miR-133 for DR of AHCFs into iCMs (19). PLGA/PEI NPs showed cytocompatibility and pH-dependent payload release and induced the expression of structural (α-sarcomeric actinin) and functional (cTnT) CM proteins at 7 days post-transfection (19). Recently, Yang et al. have reported that branched-PEI coated nitrogen-enriched carbon dots (BP-NCDs) can encapsulate miRcombo for DR of neonatal mouse CFs into iCMs *in vitro* and *in vivo* (20). BP-NCDs efficiently delivered miRNAs to target cells, inducing the expression of specific CM genes (Nppa, Nkx2.5, and Myh7) and proteins (Gata4, Mef2c, Hand2, and Tbx5) *in vitro*. Furthermore, *in situ* delivery of miRcombo by BP-NCDs in an MI mouse model significantly reduced the infarcted area after 4 weeks compared with control groups. On the other hand, limitations of this study derive from the testing of neonatal mouse CFs rather than adult human fibroblasts, the lack of extensive characterization of BP-NCDs in terms of encapsulation ability and release kinetics, and the non-degradability of the nanocarriers, which is associated with long-term safety concerns.

Hence, optimal nanocarriers for miRcombo delivery to human adult fibroblasts are currently missing. Furthermore, fibroblast specific targeting with functionalized NPs (e.g., using peptides, antibodies, or aptamers) could increase NP specificity of cargo release, thus maximizing DR effects and reducing off-target effects (21). However, specific ligands that recognize only fibroblasts populating the fibrotic scar are still under study.

## Biomimetic Culture Microenvironment

Previous literature has reported higher DR yield achieved during *in vivo* experiments in mouse model compared with *in vitro* 2D cultures. This finding suggests that a three-dimensional (3D) culture microenvironment mimicking the biophysical and biochemical properties of cardiac tissue (Table 1) has the potential to significantly improve cardiac DR outcomes (13). *In vitro* culture of miRNA-transfected fibroblasts in cardiac tissue mimetic microenvironments may enhance DR efficiency and iCM maturation, as discussed in the next paragraphs (Table 2).

**Table 1:** Reference cardiac tissue-like properties for biomimetic culture microenvironment.

**Properties**	**Reference values in cardiac tissue**	**References**
Human cardiac ECM composition	70% fibrillar collagen (collagen I and V), 20% basement membrane (collagen IV, laminin, agrin, perlecan, nidogen), 4% structural ECM (proteoglycans and fibrous glycoproteins), 3% matricellular components (collagen VI, fibronectin)	(22)
Stiffness	1–6 kPa (fetal); 10–15 kPa (adult); > 50 kPa (fibrotic)	(23)
Anisotropic ratio of stiffness	1.9–3.9	(24)
Cyclic mechanical deformation	1 Hz (in humans), 10% at early stage of diastole (stiffness: 10–20 kPa) up to 15–22 % at the end of diastole (stiffness: 50 kPa)	(24)
Electrical conductivity	0.57 S/m	(24)
Topographical cues	In native heart, myocardial fibers are arranged into distinct laminae (4–6 myocytes thick) separated by collagen-based ECM: helical-laminar assembly of hierarchically organized fibrillar structures.	(25)

**Table 2:** Selected bioengineering studies on DR of fibroblasts into iCMs.

**Cells**	**DR agents**	**Transfection agent**	**Culture substrate signaling**		**Paracrine and/or small molecules**	**Physical stimulation**	**DR efficiency**	**References**
			**Biochemical**	**Biophysical**				
Mouse neonatal and adult CFs	miRcombo	Dharmafect (*in vitro*) Lentivirus (*in vivo*)	–	TCP	Jak Inhibitor I	–	CM genes after 7 days, ~28% reprogrammed cells, calcium transients, *in vivo* cardiac recovery after MI	(6)
Mouse neonatal CFs, TTFs	miRcombo	Dharmafect™	fibrin/Matrigel™	3D hydrogel	–	–	Increased cTnT and α-sarcomeric actinin, CM gene after 15 days compared to TCP	(26)
AHCFs	miRcombo	Dharmafect™	–	TCP	–	–	CM genes, ~11% cTnT^+^ cells at 15 days, calcium transients at day 30	(7)
AHCFs	miR-1 and miR-133	PLGA/PEI NPs	–	TCP	–	–	cTnT and α-sarcomeric actinin at 7 days	(19)
Mouse neonatal CFs	miRcombo	BP-NCDs	–	TCP	–	–	CM genes and proteins, *in vivo* cardiac recovery after MI	(20)
TTFs, mouse CFs	GMT	Retrovirus	–	Surface of Matrigel-conjugated polyacrylamide hydrogels; microgroove	–	Mechanical	CM genes, striated cTnT^+^ cells at day 10, beating cells at 4 weeks (microgroove only)	(27)
MEFs	GHMT	Retrovirus	–	Surface of Matrigel-conjugated polyacrylamide hydrogels	–	–	CM genes, ~13.8% cTnT^+^ cells at 1 week, ~33% αMHC-GFP^+^ cells and beating cells at 4 weeks (8 kPa)	(28)
HNDFs	GMTHN	Plasmid transfection	Mouse CMs co-culture	Spin-coated nano-thin and nano-porous PLGA membrane	–	Electrical	CM genes, ~6.4% cTnT^+^ cells at 28 days	(29)
MEFs	OSKM (Oct4, Sox2, Klf4, and c-Myc)	Homozygous doxycycline-inducible OSKM mice	High laminin or RGD concentration	Functionalised poly(ethylene glycol) hydrogels	Jak Inhibitor I	–	~6.21% α-sarcomeric actinin^+^ cells, CM genes, beating cells at 18 days	(30)
Adult TTFs	AGHMT	Retrovirus	–	TCP	ZNF281	–	CM genes, ~33% αMHC-GFP^+^, ~45% cTnT^+^, and ~28% αMHC^+^/cTnT+ TTFs at 7 days, calcium transients, beating cells at 4 weeks	(31)
MEFs, adult CFs	Chemical cocktail (9)	–	–	TCP	PTC-209	–	~40% of MEFs and ~10% CFs αMHC^+^, cTnT and Mlc-2v, CM genes, calcium transients	(32)
MEFs, post-natal and adult TTFs	GMT, GHMT	Retrovirus	–	TCP	Diclofenac	–	CM genes, αMHC- and α-sarcomeric actinin- positive cells, calcium transients, beating cells	(33)
MEFs, TFFs	GMT	Retrovirus	–	TCP	FGF2, FGF10, VEGF	–	CM genes, calcium transient, beating cells, αMHC- and α-sarcomeric actinin-positive cells	(34)
MEFs, adult CFs	HNGMT	Plasmid	–	TCP	SB431542	–	CM genes, ~5 fold increase reprogrammed cells	(35)
MEFs, adult CFs	GHMT, miRs-1 and 133	Retrovirus	–	TCP	Y-27632, A83-01	–	CM genes, cTnT α-sarcomeric actinin- positive cells, reprogramming efficiency over 60%, beating iCMs	(16)

*This table shows differential DR strategies for iCM generation combining cell source, reprogramming factors and delivery methods, biomimetic microenvironment, paracrine signaling and/or inhibitors and physical stimuli. DR efficiency reports the expression of cardiomyocyte genes and protein, electrophysiological characters and beating property. CFs cardiac fibroblasts, MEFs mouse embryonic fibroblasts, TTFs tail-tip fibroblasts, AHCFs adult human cardiac fibroblasts, HNDFs human neonatal dermal fibroblasts, α-MHC α-myosin heavy chain, cTnT cardiac troponin T, CM cardiomyocyte, TCP tissue culture polystyrene*.

### Cell-Substrate Interactions: Biochemical and Biophysical Properties of the Culture Substrate

In 2016, Sia et al. studied DR of neonatal tail tip fibroblasts (TTFs) transfected with retroviruses expressing Gata4, Mef2c, and Tbx5 (GMT). After transfection, the cells were seeded on Matrigel-coated polyacrylamide hydrogels with different stiffness (from 1 to 62 kPa). Reprogramming yield after 10 days of culture (~17%) did not vary with substrate stiffness (27). On the contrary, microgroove culture substrates increased the yield (~30%) of fibroblasts DR into iCMs. Cells showed sarcomere structures and spontaneous contractile activity, attributed to higher expression of Mkl1, a mechanosensitive TFs, and histone H3 acetylation for chromatin remodeling (27).

More recently, embryonic mouse fibroblasts, transfected with Gata4, Mef2c, Tbx5, and Hand2 (GMTH), were cultured on Matrigel-conjugated polyacrylamide hydrogels with different stiffnesses (1–126 kPa). Higher DR efficiency was obtained on substrates with similar stiffness (8 kPa) to healthy myocardium, compared with rigid polystyrene dishes (~GPa). This result was attributed to the suppression of YAP/TAZ (Yes-associated protein/transcriptional coactivator with PDZ-binding domain) signaling and silencing of fibroblast gene programs, induced by a culture microenvironment with biomimetic stiffness (28). With respect the previous work by Sia et al. (27), the more efficient protocol for cardiac DR by GMTH transfection and the use of embryonic fibroblasts could account for the superior DR efficiency, despite the use of similar culture substrates. Although this result suggests variability of DR outcomes depending on fibroblasts types and reprogramming protocol, the role of mechanosensing was outlined. However, both studies were limited by the investigation of 2D cell cultures on the surface of hydrogels or microgroove substrates.

DR of fibroblasts embedded in 3D biomimetic matrices was only studied by Li et al. (26). In their work, miRcombo-transfected mouse fibroblasts cultured into 3D fibrin/Matrigel hydrogels showed higher DR efficiency compared with 2D cultures, as suggested by the higher expression of CM genes (α-MHC, cardiac troponin I, α-sarcomeric actinin, and Kcnj2) and proteins (cardiac troponin I and α-sarcomeric actinin). Such result was attributed to the upregulation of specific matrix metalloproteinases when cells were embedded in 3D hydrogels (26). Notably, 3D cell culture alone was sufficient to enhance the expression of CM TFs in non-transfected mouse fibroblasts compared with 2D cell cultures, suggesting that 3D culture microenvironment itself can promote the expression of CM genes (26).

Beyond biophysical characteristics of culture substrates, biochemical cues, such as proteins of the cardiac extracellular matrix (cECM), can help in recreating similar *in vitro* culture conditions to *in vivo* microenvironment (36). Indeed, in a different application, a 3D microenvironment containing brain ECM (bECM) was found to boost fibroblast DR into induced neuronal cells (iNs) (37). In this regard, gene set enrichment analysis (GSEA) of mouse embryonic fibroblasts (MEFs) transduced with MGT (Mef2c–Gata4–Tbx5) plasmids have shown that cECM proteins, such as collagens and laminins, are already expressed after 48 and 72 h post-transduction (38). Such findings suggest that in the early stages of fibroblast reprogramming, cells naturally create a suitable microenvironment, which enhances transdifferentiation. Indeed, Smith et al. have designed culture substrates based on PEG hydrogels functionalized with a high concentration of laminin and RGD peptide, achieving more efficient DR of mouse fibroblasts into iCMs, compared with hydrogels with low concentrations of RGD adhesion motifs or tissue culture polystyrene surfaces (30).

### Paracrine Signaling and Small Molecules

Fibroblast DR *in vivo* is influenced by innumerable extrinsic factors of the cardiac microenvironment, encompassing not only mechanical forces or topographical cues but also the presence of cytokines, growth factors, and paracrine signals in the heart. After MI, pro-inflammatory cytokines are released in the wounded area, supporting cardiac remodeling through immune cells and fibroblast recruitment, inducing the deposition of stiffer ECM (39). Given the key roles that cytokines play during MI, it is worth studying how cytokines may influence cell reprogramming. Enrichment analysis of pathways that regulate cardiac reprogramming showed that anti-inflammatory cytokines (IFNA2, IFNA16, and IL10) are positively associated with DR and are called “activators,” while pro-inflammatory molecules (IL1A, IL2, and IL26 cytokines and TF CEBPβ) were mostly identified as “inhibitors” (31). Indeed, TF ZNF281 was found to enhance cardiac DR via downregulation of genes involved in inflammatory response. Similarly, Testa et al. showed that treatment of mouse CFs with PTC-209, a Bmi1 inhibitor, before DR, negatively affected STAT3 and ERK1/2 phosphorylation, improving DR of fibroblasts into iCMs via inhibition of inflammatory pathways (32). Additionally, Jayawardena et al. found that JAK/STAT pathway suppression using Jak Inhibitor I, combined with miRcombo, enhanced DR of mouse fibroblasts into iCMs *in vitro* (6). Small molecule diclofenac, an inhibitor of cyclooxygenase-2 (COX-2) signaling, was also found to significantly enhance DR via PGE2/EP4 suppression, inducing sarcomere organization and increased number of beating cells as compared with GHMT alone in TTFs (33).

Moreover, a potential approach for improving DR relies also on inhibiting fibroblast endogenous signaling pathways that maintain fibroblast identity. Silencing of transforming growth factor-beta (TGF-β) and rho-associated kinase (ROCK) signaling combined with different reprogramming cocktails was reported to improve DR. The use of TGF-β inhibitor SB431542 in combination with GHMT in mouse embryonic and adult fibroblasts was reported to induce ~5-fold increase in cell reprogramming after 10 days of culture (35). Moreover, Zhao et al. have reported that DR is enhanced in GHMT-transfected mouse fibroblasts by overexpressing miR-1 and miR-133 combined with ROCK or TGF-β inhibitors, suggesting a synergistic effect in overcoming reprogramming barriers (16).

Yamakawa et al. have studied MEF reprogramming into iCMs in defined serum-free medium containing fibroblast growth factor (FGF) 2, FGF10, and vascular endothelial growth factor (VEGF) (34). The addition of these growth factors after cell transduction with Mef2c and Tbx5 successfully generated iCMs, exhibiting calcium oscillation and spontaneous contraction, by activating cardiac transcriptional regulators, including Gata4. Defined culture conditions influenced cardiac DR only in the later stage of transdifferentiation (34).

### Further Physical Stimulations: Cyclic Mechanical Stretching and Electrical Stimulations

Mechanical stimulation of cultured induced pluripotent stem cell (iPSC)-derived CMs was found to enhance cellular alignment and sarcomere organization, calcium handling, and contractile properties, causing alterations in gene and protein expression toward a mature phenotype (40). As described above, Sia et al. have investigated different biophysical stimuli to induce fibroblast DR. Mechanical cyclic stimulation (10% strain, 1-Hz frequency) applied for 10 days reduced the percentage of reprogrammed cells compared with static cultures (27). Under mechanical stimulation, the expression of hallmarks of fibrotic scar tissue (collagen I and fibronectin) and reinforcement of fibroblast signature could account for the detected decrease in DR yield (41). On the other hand, mechanical cyclic stretching applied at a later stage of cardiac DR could potentially improve iCM maturation, as suggested by the wide literature on iPSC differentiation into CMs (42).

Electrical stimulation was also tested in cardiac DR, considering its beneficial effect on maturation of stem cell-derived CMs (43). Heart-like electric stimulation (1 V/cm, biphasic square pulse for 5 ms at 5 Hz) of GMTHN (Gata4, Mef2c, Tbx5, Hand2, and Nkx2.5) transfected human neonatal dermal fibroblasts (HNDFs), cocultured with murine CMs on nano-thin and nano-porous PLGA membranes, significantly increased DR yield, inducing the expression of CM genes and increasing the percentage of cTnT-positive cells (29). Additionally, cardiac cell sheets formed by reprogrammed cells were implanted in infarcted hearts, leading to cardiac function improvements and decreased adverse cardiac remodeling post MI (44). Although wider investigation is needed, such early studies suggested the positive role of electrical stimulation on DR efficiency.

## Discussion

Nowadays, MI still remains one of the leading causes of death worldwide. Hence, strategies for the replacement of CM loss are of primary interest in regenerative medicine.

DR of human fibroblasts into iCMs might represent a new therapeutic option for myocardial regeneration in addition to cell therapies with iPSC-CMs. Indeed, iPSC-CMs can now be obtained with high efficiency, although their maturation level is generally low, resembling fetal stage CMs (45). Finally, therapies using cells differentiated from pluripotent stem cells, such as iPSCs or embryonic stem cells (ESCs), suffer from the risk of teratoma formation (46).

DR could be exploited as a new source for autologous CMs derived from trans-differentiation of patients' fibroblasts with the advantage of low-to-null tumorigenicity risk if obtained by non-viral methods (9, 20). Current research efforts are addressed to increase DR yield and to approach a more adult iCM phenotype. However, one disadvantage of iCMs use in cell therapy is the need for high amounts of patients' fibroblasts (in which potential for *in vitro* expansion is reduced with respect to stem cells) to generate the required quantities of CMs (from tens to hundreds of millions).

With respect to iPSC-derived technologies, miRcombo-mediated DR also paves the way to new cell-free *in situ* strategies for cardiac regeneration, based on the local injection of reprogramming agents able to induce DR of CFs of fibrotic areas or their boundaries into iCMs. Non-viral approaches for *in situ* DR are safer than viral vectors, in which use is limited by possible off-target effects, mutagenesis risk for integrative virus (retrovirus and lentivirus), and strong immune response (47). Among non-viral strategies for cardiac DR, *in vivo* administration of small molecule combinations is complicated by the need to locally treat CFs with many drugs (up to nine small molecules) at specific relative concentrations (48, 49). On the contrary, the approach based on transient transfection of fibroblasts with miRcombo requires efficient simultaneous release of four miRNAs (miR-1, miR-133, miR-208, and miR-499) to CF cytoplasm. For efficient *in vivo* DR, nanocarrier surface could be functionalized with selected ligands for CF recognition, coupled to anti-fouling molecules (e.g., ethylene glycol oligomers) to ensure drug delivery specificity *in vivo*. Additionally, studies on optimal miRcombo dose and delivery kinetics into fibroblast cytoplasm are missing, while it would be fundamental to design efficient miRcombo delivery systems.

Although arrhythmic events have not been reported by reports on *in situ* cardiac DR in mouse models, this risk could potentially arise from the initial immaturity of early iCMs and be minimized by reducing DR time needed for effective CFs DR into mature iCMs (50). Additionally, based on very recent findings on iPSC-CMs therapies, administration of antiarrhythmic drugs could be considered and studied to assist early DR phases, enhancing patients' safety (51).

Another important feature of cardiac DR is its decreasing efficiency as a function of fibroblast aging (52). Scientific literature reported that embryonic vs. adult fibroblasts have higher chances for conversion due to an open chromatin conformation (52). However, most DR studies employed embryonic, fetal, or post-natal mouse fibroblasts with superior transdifferentiation ability (52). Importantly, *in vitro* studies with mouse cells also provide specie-specific outcomes with limited relevance and predictivity for humans. As an exception, pig CFs might be employed considering their close features to human CFs (53). However, investigation on AHCFs is preferred in the perspective of future clinical translation of the approach. In this regard, DR is affected by “patient specificity,” as its efficiency may vary significantly, depending on patients' age, sex, and genetic background. Overall safe and efficient standardized protocols taking into account patients' specificity should be defined, allowing more efficient cardiac DR based on clinical cases (52).

Currently, DR research is still at its basic steps. Hence, thorough *in vitro* studies are demanded, elucidating the role of biochemical and biophysical factors on DR efficiency of AHCFs into iCMs. Based on early findings (26), understanding and controlling the biochemical and biophysical cues of 3D culture substrates are the key for the design of instructive microenvironments improving DR efficiency and fostering the generation of mature iCMs (Table 2). Optimal 3D substrates should mimic cardiac tissue-like stiffness, composition, and architecture (Table 1). However, cell remodeling progressively alters the composition, permeability, and stiffness of 3D culture matrices, providing dynamic spatiotemporal cues affecting cell fate (54). New advanced techniques able to monitor 3D cell cultures could unravel the effects of dynamic microenvironmental changes on DR outcomes (55, 56). Such interdisciplinary research could be beneficial for efficient DR of human adult fibroblasts into iCMs, given their high epigenetic resistance to phenotype switch. Properly selected types and doses of anti-inflammatory and anti-fibrotic soluble factors could also help in suppressing fibroblast signature, to address the intrinsic epigenetic resistance of adult fibroblasts. Furthermore, more in-depth investigations of the effects of mechanical and electrical stimulations on DR yield and iCM maturation are still rather limited and deserve future attention.

## Conclusions

The discovery of key biochemical and biophysical factors enhancing cardiac DR and the design of effective and safe nanocarriers for targeted miRcombo delivery will result in significant progresses of both *in vitro* and *in situ* cardiac DR approaches, fostering technological advances toward the future clinical application of cardiac DR strategies. However, full exploitation of DR potentialities requires an intense interdisciplinary research, in which bioengineering studies play a key role for the full exploitation of the potentialities of this new emerging approach.

## Author Contributions

The manuscript was conceived and written by CP and VC. VC supervised the project and acquired the funding. Both authors have given approval to the final version of the manuscript.

## Funding

This work has received funding from the European Research Council (ERC) under the European Union's Horizon 2020 Research and Innovation Programme (Grant Agreement No. 772168), through BIORECAR ERC Consolidator project (www.biorecar.polito.it).

## Conflict of Interest

The authors declare that the research was conducted in the absence of any commercial or financial relationships that could be construed as a potential conflict of interest.

## Publisher's Note

All claims expressed in this article are solely those of the authors and do not necessarily represent those of their affiliated organizations, or those of the publisher, the editors and the reviewers. Any product that may be evaluated in this article, or claim that may be made by its manufacturer, is not guaranteed or endorsed by the publisher.
